# Merkel Cell Polyomavirus: Molecular Insights into the Most Recently Discovered Human Tumour Virus

**DOI:** 10.3390/cancers6031267

**Published:** 2014-06-27

**Authors:** Gabrielė Stakaitytė, Jennifer J. Wood, Laura M. Knight, Hussein Abdul-Sada, Noor Suhana Adzahar, Nnenna Nwogu, Andrew Macdonald, Adrian Whitehouse

**Affiliations:** School of Molecular and Cellular Biology and Astbury Centre of Structural Molecular Biology, University of Leeds, Leeds, LS2 9JT, UK; E-Mails: bsgst@leeds.ac.uk (G.S.); bsjjw@leeds.ac.uk (J.J.W.); bslmk@leeds.ac.uk (L.M.K.); bshkas@leeds.ac.uk (H.A.-S.); bsnsba@leeds.ac.uk (N.S.A.); bsnoun@leeds.ac.uk (N.N.); A.Macdonald@leeds.ac.uk (A.M.)

**Keywords:** Merkel cell polyomavirus, Merkel cell carcinoma, large T antigen, small T antigen, tumourigenesis

## Abstract

A fifth of worldwide cancer cases have an infectious origin, with viral infection being the foremost. One such cancer is Merkel cell carcinoma (MCC), a rare but aggressive skin malignancy. In 2008, Merkel cell polyomavirus (MCPyV) was discovered as the causative agent of MCC. It is found clonally integrated into the majority of MCC tumours, which require MCPyV oncoproteins to survive. Since its discovery, research has begun to reveal the molecular virology of MCPyV, as well as how it induces tumourigenesis. It is thought to be a common skin commensal, found at low levels in healthy individuals. Upon loss of immunosurveillance, MCPyV reactivates, and a heavy viral load is associated with MCC pathogenesis. Although MCPyV is in many ways similar to classical oncogenic polyomaviruses, such as SV40, subtle differences are beginning to emerge. These unique features highlight the singular position MCPyV has as the only human oncogenic polyomavirus, and open up new avenues for therapies against MCC.

## 1. Introduction

The causes of cancer are many and varied, but viruses and other infectious agents are being recognised as increasingly important. The International Agency for Research on Cancer estimates that around 20% of cancer cases worldwide are caused by infection, with most being viral in origin [1]. Today, seven human oncogenic viruses are thought to account for 10%–15% of human cancers.

Oncogenic viruses can cause cancer by interrupting the control of cell proliferation. Most express specific oncogenes that promote cell growth and proliferation, thus transforming cells. The first oncogenic virus, Rous sarcoma virus or RSV, was discovered by Peyton Rous in 1911, and causes cancer in chickens [2]. In the following decades, a series of mammalian oncogenic viruses were discovered, including murine polyomavirus (MPyV) and simian vacuolating virus 40 (SV40) [3,4,5]. The latter have served as an excellent model for both studying oncogenic viruses and fundamental oncogenic processes.

Human oncogenic viruses have been the focus of intensive study, ever since the discovery of Epstein-Barr virus (EBV) in 1964 [3]. They represent a variety of different types of viruses, from small DNA viruses to RNA viruses to retroviruses, representing the range of possible viral life cycles and modes of reproduction. Table 1 elaborates on these viruses and their links to cancers.

**Table 1:** The human oncogenic viruses. The seven human oncogenic viruses, their year of discovery, genome and various cancers they are associated with. ds-double-stranded, ss-single-stranded, (+)-positive-stranded.

VIRUS	YEAR	FAMILY	GENOME	ONCOGENES	CANCER ASSOCIATION
Epstein-Barr Virus (EBV)	1964	Herpesviridae	dsDNA	LMP1	Including Burkitt’s lymphoma, nasopharyngeal carcinoma, and some other lymphoproliferative disorders
Hepatitis B virus (HBV)	1965	Hepadnaviridae	ssDNA and dsDNA	HBx	Some hepatocellular carcinomas
Human T-lymphotropic virus-I (HTLV-I)	1980	Retroviridae	ssRNA	Tax	Adult T cell leukaemia
Human papillomaviruses (HPV) 16 and 18	1983-1984	Papillomaviridae	dsDNA	E5, E6, E7	Most cervical cancer and penile cancers.
Hepatitis C virus (HCV)	1989	Hepaciviridae	(+)ssRNA	NS5A	Some hepatocellular carcinomas and lymphomas
Kaposi’s sarcoma herpesvirus (KSHV)	1994	Herpesviridae	dsDNA	LANA, vflip, and vBcl-2, among others	Kaposi’s sarcoma and primary effusion lymphoma
Merkel Cell Polyomavirus (MCPyV)	2008	Polyomaviridae	dsDNA	T antigens	Most Merkel cell carcinomas,

The newest addition to the human oncogenic virus classification is Merkel cell polyomavirus (MCPyV), discovered in 2008 [6]. It has been confirmed to be the causative agent behind the majority of cases of Merkel cell carcinoma (MCC), a rare and highly aggressive human malignancy.

## 2. Merkel Cell Carcinoma

Merkel cell carcinoma is a rare and highly malignant primary neuroendocrine carcinoma of the skin. Over 90% of cases arise in sun-exposed areas, with half around the head and neck. This suggests that sunlight, primarily as ultraviolet radiation, plays a role in the development of MCC. MCC can also be found on the trunk and genitals but at a significantly reduced frequency (<10%) [7]. Epidemiological studies highlight that older, lighter-skinned individuals and people who have been subjected to organ transplant or infected with HIV/AIDS are more vulnerable to infection [8,9].

**Figure 1:**
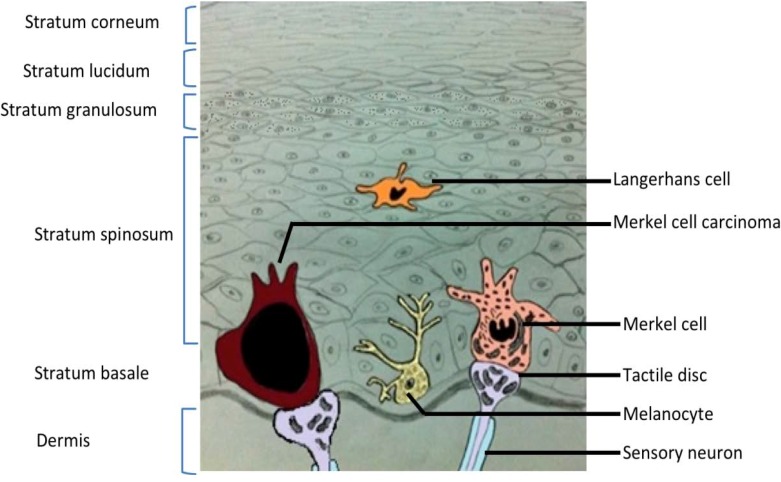
Cross section of skin. Illustration of the six layers of skin, from the dermis to the stratum corneum. Examples of residents cells are included, among them a healthy Merkel cell and a cell that is part of a Merkel cell carcinoma tumour. Merkel cells are located in the stratum basale.

MCC is thought to arise in Merkel cells, a type of mechanoreceptor cell located in the stratum basale in touch sensitive areas of the epidermis (Figure 1). Merkel cells were first described over a century ago by the leading German histopathologist Friedrich Sigmund Merkel [10]. These cells are oval and around 10–15 µm in diameter, resembling a hemisphere with spinous projections on their surfaces. They are found attached to the disk-like sensory terminal of nerve fibres, in a complex termed a Merkel or tactile disk [11]. The function of Merkel cells is still unclear. Studies indicate that the presence of neuropeptides, accumulated near the nerve fibre junction, signifies their action in the expression of voltage gated ion channels which are essential for synaptic transmission. However, conflicting evidence, using histochemical markers, suggests a paracrine function of Merkel cells [12].

**Figure 2:**
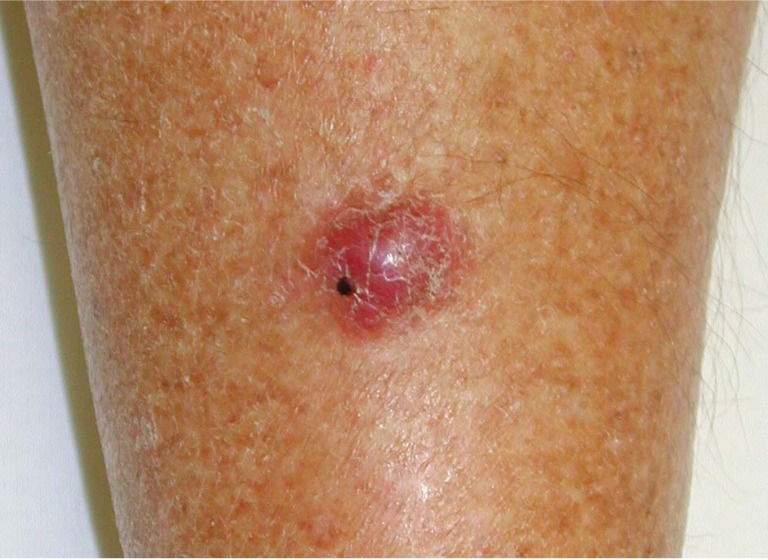
Merkel cell carcinoma. A typical MCC nodule present on the leg of a patient. Image credit to Howard Peach, Leeds Teaching Hospitals NHS Trust, UK.

MCC was first described in 1972 by Cyril Toker [10], who noted a flesh-coloured or bluish-red glassy painless nodule (Figure 2) with solid trabeculae in five different areas (forearm, lip, face, leg and buttock) of two older men, who later died as a result of this tumour, and three older women. Toker used the name trabecular carcinoma of the skin to describe these tumours, assuming that they represented an endocrine, sweat gland-derived carcinoma.

Advanced age (>65 years) and male gender correlate with the development of MCC [13]. The estimated annual incidence of MCC has increased during the last decade from 0.29 to 2.2 cases per million, and more than half of infected patients die within 5 years of diagnosis. Though the mortality rate of MCC is much higher than those for other skin cancers [14], this may in part be due to the increased incidence of secondary cancers in patients diagnosed with MCC, such as chronic lymphocytic leukaemia, multiple myeloma, malignancies of the biliary tract and salivary gland, and other skin malignancies, such as basal cell carcinoma [15]. MCC is a highly metastatic tumour and can transplant to other parts of the body by transiting through the lymphatic drainage [16]. MCC metastasis have often been reported at distant sites including the pancreas and breast [17], the heart [18], and the palatine tonsil [19].

## 3. Merkel Cell Polyomavirus

MCPyV is a double-stranded DNA virus of the *Polyomaviridae* family. It was discovered in 2008 by Moore and Chang at the University of Pennsylvania, using digital transcriptome subtraction (DTS), a novel method of detecting viruses in cancerous tissue. DTS involves extracting mRNA from tumor cells, converting it to cDNA, and then sequencing. The data obtained is then compared to known human sequences, and matching stretches of DNA are “subtracted”, leaving the remainder as likely non-human. The remaining sequences are then compared against the pathogen sequence database. The analysis highlighted a sequence related to polyomaviruses, but distinct enough to be considered a novel virus [6].

Initially, the MCPyV genome was found integrated into the genome of MCC cells in a clonal pattern [6]. Moreover, based on the fact that MCPyV infection and genome integration happens before the clonal expansion of tumor cells suggests that MCPyV might be a contributing factor to MCC. The mechanism by which MCPyV causes tumorigenesis is still questionable. Following initial findings, several other reports confirmed clonal integration of the viral genome into the cellular genome in MCC tumors [20,21,22]. These reports support the theory that MCPyV is an etiological factor of MCC, rather than being a passenger virus. In addition, MCC tumour metastases were also found to be MCPyV-positive and possess the same integration patterns as the original tumour [20]. MCPyV is the only polyomavirus that has been conclusively associated with a human tumor, and has now been detected in 80%–97% of MCC tumors [6,23]. 

All MCC cells display membrane-bound cytoplasmic structures with a unique arrangement of cytokeratin 20 (CK20) filaments in a perinuclear distribution [24]. This feature is used to distinguish and diagnose MCC. The primary diagnostic panel of MCC also includes the thyroid transcription factor and leukocyte common antigen. Moreover, the presence of chromogranin and synoptophysin confirm the positive diagnostic results of MCC.

In addition, immunohistochemistry has been used to detect MCPyV viral antigens in MCC tumours, with antigen expression being limited to MCC cells [25]. More recent serological assays that detect MCPyV VP1 and VP2 proteins by enzyme-linked immunosorbent assay (ELISA) of blood sample have made the diagnosis of MCPyV easier [26]. These depend on the lack of similarity between the MCPyV VP1 and the capsid protein of other polyomaviruses. These assays have been used to determine the incidence of MCPyV in the general population, as well as giving clues about its epidemiology. MCPyV shows a worldwide distribution, and has been detected in samples from the United States [27], Europe [28], Japan [29], Cameroon in Africa [30] and Australia [31], among others. This highlights the startling fact that not only are the majority of MCC patients positive for the virus, but in the wider population, 50% of children under 15 and up to 80% of healthy adults are infected with MCPyV, as reflected by seroprevalence [27]. It is likely that MCPyV is acquired at some point during childhood, either by transmission between siblings or from mother to child, through close contact, involving skin and/or saliva [30]. In addition, recent evidence has suggested that there are geographical variations of MCPyV [32,33], with the possibility of as many as five different genotypes present across the globe [34]. The virus is considered a common skin commensal, and further work is bound to uncover more details of its epidemiology and genotypical variations.

## 4. MCPyV Genome

**Figure 3:**
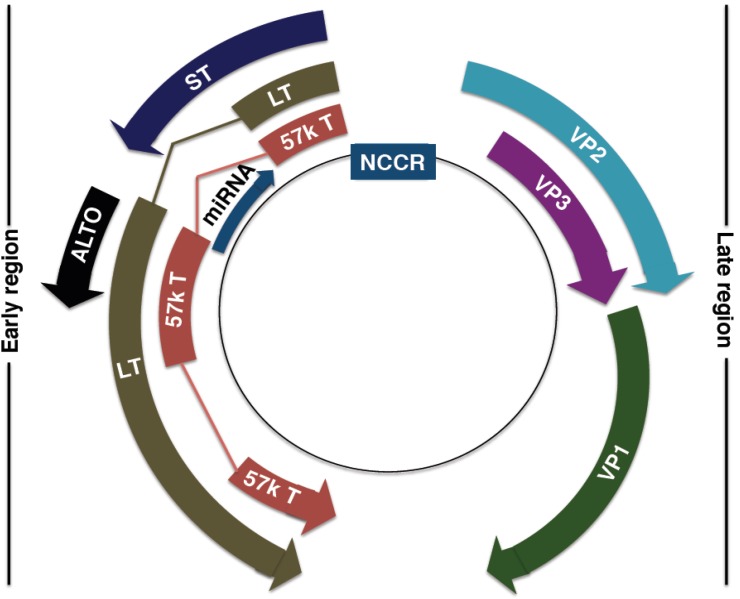
MCPyV genome organisation. Non-coding control region (NCCR): bipartite origin of replication. Early gene region: Large T antigen (LT), small T antigen (ST), 57kT antigen (57kT), alternative T antigen open reading frame (ALTO), microRNA (miRNA). Late gene region: capsid proteins (VP1-3).

The MCPyV genome (Figure 3) comprises 5,387 base pairs, with a non-coding control region (NCRR), containing the bipartite origin of replication, separating the early and late gene coding regions. Separation allows for temporal control of viral gene expression. This type of genome structure is closely related to that of all known polyomaviruses. While there is much shared homology between MCPyV and other well-studied human polyomaviruses, the MCPyV genome seems to be most closely related to the African Green Monkey lymphotropic polyomavirus [35]. Currently, MCPyV is classified in the Orthopolyomavirus genus of the *Polyomaviridae* family, which include mammalian polyomaviruses such as MPyV, SV40 and the human polyomaviruses JC (JCPyV) and BK (BKPyV) [36].

### 4.1. MCPyV Origin of Replication

The NCCR contains the 71 base pair long origin of replication. The origin of replication, like those of other polyomaviruses, has an AT rich region, an early enhancer domain and a binding site for the large T antigen. The binding site is composed of ten repeating guanine-rich pentanucleotide sequences: eight corresponding to the general polyomavirus consensus of 5'-GAGGG-3', while two are somewhat different: 5'-GGGGC-3' and 5'-GAGCC-3'. The number of sequences and their proximity to each other are unlike other polyomaviruses [37].

### 4.2. MCPyV T Antigen Locus

**Figure 4:**
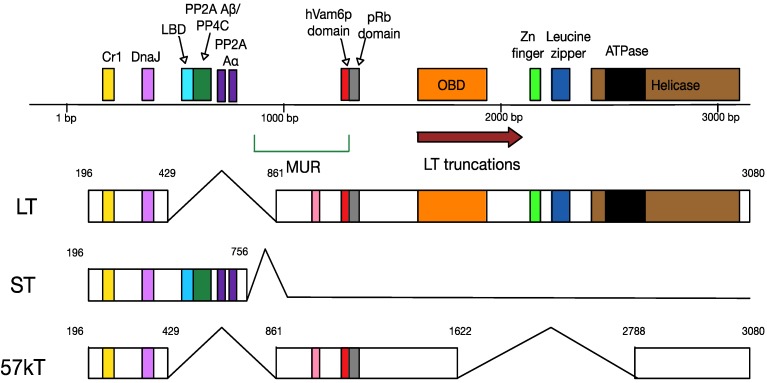
Mapping of the multiply-spliced MCPyV T antigens. The three T antigens are LT, ST and 57kT. All three encode CR1 (yellow, LXXLL) and DnaJ (lilac, HPDKGG) domains. ST also contains two PP2A Aα binding sites (R7 and L142), a PP2A Aβ/PP4C binding site (amino acids 97–111) and an large T-stabilisation domain (LSD, amino acids 91–95). LT shares the pRb binding domain with 57kT; in addition, it has unique origin binding (OBD), zinc finger, leucine zipper, ATPase and helicase domains. The MCPyV-unique region (MUR) of LT contains the hVam6p binding site.

Polyomavirus early gene regions encode proteins that interfere in cell cycle regulation, and, in some cases, lead to tumour formation. For this reason, the polyomavirus early gene region is also known as the “tumour antigen” or T antigen locus (Figure 4). The MCPyV T antigen locus is about 3000 base pairs in length. After transcription as a single transcript, the T antigen is differentially spliced into the large T antigen (LT), the small T antigen (ST), and the 57 kD T antigen (57kT) [6]. LT, ST and 57kT all share the same short amino-terminal sequence, including the J domain. This contains the DnaJ (HPDKGG) domain, which is essential for binding the cellular heat shock protein, Hsc70, and a conserved Cr1 epitope (LXXLL) [6]. Alternative splicing produces a distinct carboxy-terminus, which determines the variability in the functions of the T antigen splice products.

MCPyV LT, 816 amino acids in size, is composed of two exons. It has numerous functions in MCPyV infection, including the initiation of viral replication and manipulation of the host cell cycle. MCPyV LT contains several conserved domains found across polyomaviruses: the retinoblastoma protein (pRb) binding domain (LXCXE), a nuclear localisation signal (NLS) and the origin-binding domain (OBD). The carboxy-terminal half of the polyomavirus LT has viral DNA binding and helicase features [38,39,40,41]. The presence of a nuclear localisation signal (NLS) results in MCPyV LT localising to the nucleus when expressed in mammalian cells [42].

It may be possible to infer some interactions between MCPyV LT and cellular factors by considering SV40 LT. The DnaJ domain of SV40 LT interacts with the cellular chaperone Hsc70, and the LXCXE domain binds to pRb and pRb family members p107 and p130 [43,44]. The E3 ligase component Cullin 7 and the spindle checkpoint protein BUB1 are cellular factors which have been demonstrated to bind between these two regions [45,46]. The OBD and helicase region recruit numerous proteins required for viral replication, including p53, p300 and CBP [47,48]. However, MCPyV LT shares only 34% sequence identity with SV40 LT [40]. MCPyV LT also contains the MCPyV unique region (MUR), consisting of a 200 amino acid sequence located between the first exon and the OBD [42]. This unique LT region binds the host cellular factor Vam6p and redistributes it to the nucleus, in addition to encoding a viral miRNA [42,49].

MCPyV 57kT, 432 amino acids in length, is the product of alternative splicing linking three exons. Little is known about this protein, although it shares the DnaJ domain and CR1 epitope with LT and sT, as well as containing the MUR region and the pRB-binding domain. In addition, 57kT is homologous with SV40 17kT [50], which plays a role in promoting host cell proliferation [51].

Finally, MCPyV ST is 186 amino acids long, and, like LT, has roles in viral replication and cellular transformation. The unique carboxy-terminal ST region is produced via transcriptional read-through of the exon splice site used by both LT and 57kT. ST localises both to the nucleus and the cytoplasm [52]. In addition, it contains the typical polyomavirus protein phosphatase 2A (PP2A) Aα subunit binding site [6], which is important for viral replication and virus-induced transformation in other polyomaviruses [53]. However, recently, a PP2A Aβ and/or protein phosphatase 4C (PP4C) binding site near its carboxy terminus has also been discovered, which may have a role to play in protecting MCPyV from the cellular immune response [54]. In addition, another newly-discovered MCPyV ST domain is the LT-binding domain (LBD), which stabilises LT and aids in the replication of the MCPyV genome [55].

In addition to the three T antigens, the MCPyV early gene locus also encodes a fourth protein, the alternative T antigen open reading frame (ALTO). ALTO is transcribed from the 200 amino acid MUR region of LT, and seems to be evolutionarily related to the middle T antigen of the murine polyomavirus [56]. 

### 4.3. MCPyV Late Proteins

The structural proteins for the virus capsid are encoded in the late region. There are three open reading frames for capsid proteins, viral proteins 1, 2, and 3 (VP1, VP2 and VP3), which is typical of all polyomaviruses. However, the VP3 open reading frame is not conserved and the protein is inactive, not part of the capsid, or not expressed at all [57].

MCPyV has an unenveloped, icosahedral capsid made up of 72 pentamers of the major capsid protein, VP1. VP2, the minor capsid protein, is important for infectivity, while the presence of VP3 has not been detected in native MPCyV virions [57]. Despite the apparent absence of VP3, the viral capsid is about 40–55 nm in size, which is comparable to other polyomaviruses. The ratio of VP1:VP2 seems to be about 5:2, which means there are two VP2 molecules for each VP1 pentamer [57].

The crystal structure of VP1 was determined as a symmetrical, ring-shaped homopentamer with the five monomers arranged around a central five-fold axis. Each VP1 monomer is composed of two antiparallel β sheets, which form a β-sandwich with jelly-roll topology. Variable loops create unique interaction surfaces on the outer surface of the pentamer [58]. VP1 has a nuclear-localisation signal (NLS) at its amino-terminus, and shows a diffuse nuclear pattern.

VP2 seems to be dispensable for most entry steps in some cell lines, as it does not affect trafficking, viral DNA packaging, or binding to cellular receptors. However, it is possible that myristoylation of VP2, which is required during entry, may help to disrupt cellular membranes. VP2 seems to lack a NLS, and thus localises in the cytoplasm. However, presence of VP1 shifts the signature of VP2 to a similar, diffuse nuclear pattern [57].

### 4.4. MCPyV MicroRNA

Numerous polyomaviruses, including SV40, JCV and BKV, encode miRNAs which regulate early viral transcript levels [59,60]. It is therefore unsurprising, that in addition to the structural proteins, the late region also encodes a 22-nucleotide-long microRNA (miRNA), MCV-miR-M1-5p. The miRNA is encoded antisense to the LT coding region [49]. It is thought to regulate the expression of the early genes, reducing the levels of early gene transcripts, and has complementarity with a section of the LT transcript. Furthermore, it may have a role to play in cellular transformation, and its expression is preserved in at least half of MCPyV-positive MCC tumours [61].

Therefore, the MCPyV genome shares a many similarities in its organisation and gene products with other polyomaviruses. However, there are unique characteristics, and the reasons for these divergences are not yet fully understood.

## 5. The Lifecycle of MCPyV

The cellular tropism of MCPyV has not yet been established, as it was discovered within a malignancy, rather than its natural host cell. However, the origin of Merkel cell carcinoma as well as the findings that MCPyV is chronically shed from the skin [62], point to an epidermal source of host cells. MCPyV pseudovirions exhibit infectious entry into human skin-derived primary keratinocytes (HEKa) and transformed melanocytes, while primary transformed keratinocytes (HeCat) and primary melanocytes did not permit infection. In addition, none of the 60 human tumour cell lines investigated show tropism for MCPyV [63]. It is possible to propagate the virus in human embryonic kidney cell-derived cultures (HEK-293), when LT and ST are expressed *in trans* [64], and this is the presently used to study the life cycle of MCPyV.

### 5.1. MCPyV Attachment and Entry

As a rule, most polyomaviruses, such as MPyV, SV40, and BKPyV use sialic acid-containing glycolipids, or gangliosides, to attach to cells [65,66]. However, in the case of MCPyV, the initial attachment appears to involve glycosaminoglycans, in particular heparan sulfate. In addition, binding to gangliosides, specifically Gt1b, which carries three different sialic acids, is required for post-attachment entry [64]. 

**Figure 5:**
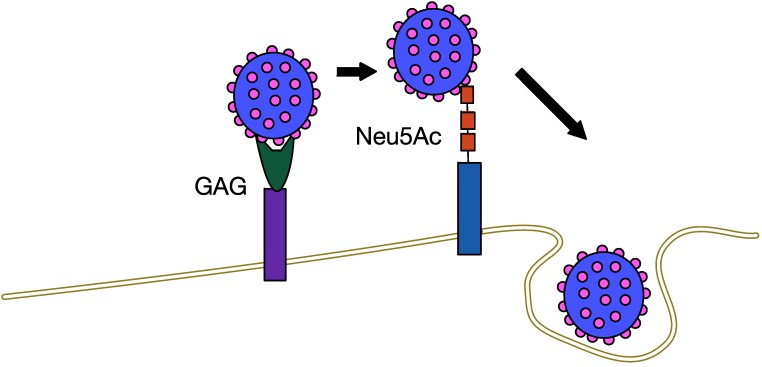
Two-step attachmentandentry process of MCPyV. GAG—glycosaminoglycan, such as heparan sulfate. Neu5Ac—ganglioside with a linear Neu5Ac-α2,3-Gal motif.

Using X-ray crystallography, researchers have been able to determine that the MCPyV major capsid protein VP1 has a shallow binding site for cellular glycans containing sialic acid with a Neu5Ac moiety. Specifically, it interacts with carbohydrates bearing a linear Neu5Ac-α2,3-Gal motif. Mutating these binding sites has no effect on initial attachment [58]. Therefore, the current model of MCPyV entry (Figure 5) is a two-step attachment-and-entry process involving two separate types of host cell plasma membrane factors. 

The utilisation of glycosaminoglycans by MCPyV for cellular attachment is reminiscent to the entry tactics of papillomaviruses, which are exclusively tropic for keratinocytes, a type of epithelial cell. This is a possible example of convergent evolution, and further suggests the epidermis as playing host to MCPyV. 

### 5.2. MCPyV Replication

Under typical conditions, in permissive cells, MCPyV is able to complete its replication cycle in the cell nucleus and form virions, without inducing tumourigenesis. As a small double-stranded DNA virus, it relies heavily on host factors to transcribe its genome and replicate. However, as in other polyomaviruses, the T antigens are vital for this purpose, and are transcribed immediately upon entry into the nucleus of the host cell. These genes induce the cell to enter S-phase, altering the cellular environment to be hospitable to viral replication. Once sufficient levels of the T antigens are present, it is possible that the miRNA encoded by the MCPyV genome inhibits further early gene transcription, and thus shifts the focus to viral replication and the transcription of the late region capsid proteins [49].

Polyomaviruses require expression of the LT antigen for initiation of genome replication. Prior to SV40 replication, LT must oligomerise to form hexameric molecules, two of which bind to the origin of replication in a head to head orientation [67]. The LT helicase domain is then responsible for unwinding DNA and replication proceeds in a bidirectional manner, with hexamers moving in opposite directions. As mentioned, the MCPyV origin of replication maps to a 71 bp minimal core region composed of a poly(T) rich tract and eight GAGGC-like pentanucleotide sequences [37]. The LT OBD interacts with the origin region via recognition of these GAGGC-like motifs. A crystal structure of the origin revealed the requirement for three of these pentanucleotide sequences for initiation of replication [68]. The number and proximity of LT binding sites on the origin likely allows for intermolecular OBD-OBD interactions between molecules of LT [37].

Several viral and cellular factors have been demonstrated to affect LT mediated replication. The cellular vacuolar sorting protein, hVam6p, binds to LT through a domain adjacent to the Rb binding site in the MUR region of the protein [42]. hVam6p is involved in late endosome and lysosome fusion as part of the homotropic fusion and protein sorting (HOPS) complex. This interaction promotes translocation of hVam6p from the cytoplasm to the nucleus, disrupting lysosome clustering. Overexpression of hVam6p was shown to reduce MCPyV particle production by over 90%, whereas mutation of the LT hVam6p binding domain increased virion production by 4 to 6 fold [69]. This indicates an inhibitory role for hVam6p, the significance of which remains unclear, although it may have to do with diminishing viral replication and establishing a persistent infection.

Another cellular factor that plays a role in MCPyV replication is the chromatin-associated bromodomain containing protein 4 (Brd4), a member of the BET protein family. Brd4 interacts with LT, acting to recruit the cellular replication factor C (RFC) to MCPyV replication complexes. RFC is needed for the loading of the PCNA clamp and the tethering of the processive DNA polymerase δ to allow for the elongation of viral DNA [70]. Expression of a dominant negative inhibitor of Brd4 abrogates viral replication *in vitro* and *in vivo*, indicating a critical role for this interaction in the MCPV life cycle. Recently, it has been shown that host DNA damage response (DDR) factors are also involved in MCPyV replication, as they co-localise to the replication foci when LT is present and active. Both the ATM- and ATR-mediated pathways seem to be involved [71]. DDR factor [72] and Brd4 [73] activation and recruitment have been observed in HPV replication, suggesting a similar suite of host factors involved in MCPyV replication.

While LT is necessary for the replication of MCPyV DNA, on its own, it does not facilitate the process efficiently. ST is needed to enhance replication, with knockdown of ST leading to inhibition of replication [37]. One way ST could play a role is by promoting the hyperphosphorylation of the translation regulator eIF4E binding protein (4E-BP1) [74], which leads to an increase in the production of cellular proteins, including host factors necessary for viral replication. In addition, ST prevents the turnover of LT by targeting the cellular SCF^Fbw7^E3 ligase. This cellular complex, of which Fbw7 is the recognition component [75], targets LT for proteasomal degradation. Fbw7 is important in cancer studies and it is deregulated in multiple cancers [76,77], with loss of Fbw7 resulting in tumourigenesis and genetic instability [78,79]. Degradation of LT can be reversed by adding the proteasome inhibitor MG132. However, binding of ST to SCF^Fbw7^ via its LT-stabilisation domain (LSD) inhibits LT degradation. In the absence of ST, the half-life of LT is 3–4 hours, while co-expression of the two proteins increases the half-life of LT to 24 hours [55]. The requirement of MCPyV ST in efficient viral replication is in stark contrast to SV40, as SV40 ST co-expression has minimal effect on SV40 LT mediated genome replication and SV40 ST is unable to cross-enhance MCPyV LT-induced viral replication despite having conserved PP2A and Hsc domains [55]. The shared DnaJ domain, which binds to the heat shock protein Hsc70, a feature important in polyomavirus replication is absolutely necessary for LT function, but is not essential for ST. Finally, neither ST nor the 57kT proteins are sufficient for replication on their own, and co-expression of 57kT with LT shows no increase in replication efficiency [37].

### 5.3. MCPyV and the Immune Response

Innate immunity is a critical intracellular barrier against invading microbes, thus it is unsurprising that pathogens commit a large proportion of their resources to evading it. Until very recently, little was known about how MCPyV responds to threats from its host cell. However, recent literature has highlighted an intriguing role of MCPyV T antigens in subverting the innate immune response to allow the establishment of early and persistent viral infection.

**Figure 6:**
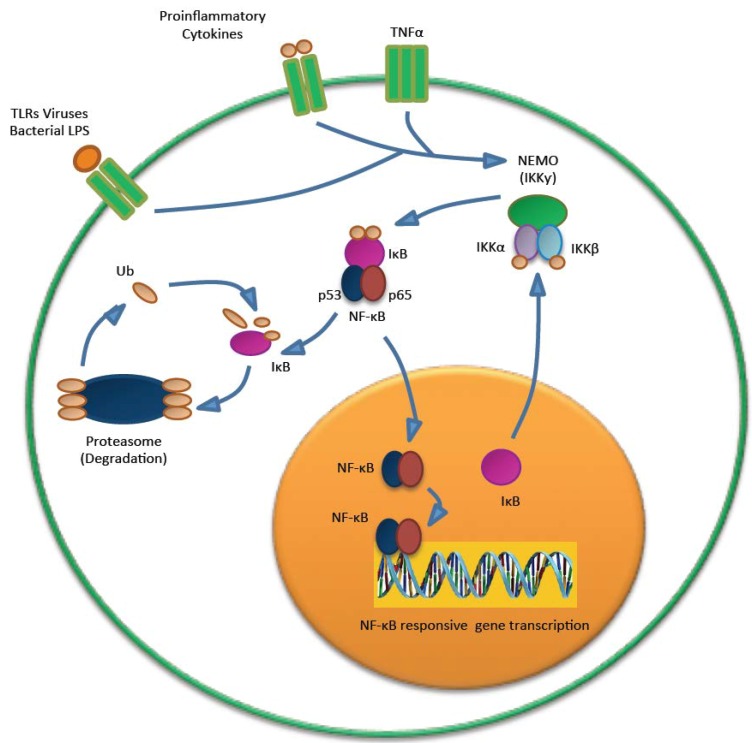
NF-κB signaling. The IKK is activated after PAMP recognition by PRRs. This leads to the proteasomal degradation of IκB and release of NF-κB, which can then translocate into the nucleaus and activate the transcription of genes that have functions in the innate immunity.

MCPyV ST has been found to play a role in downregulating the innate immune response through interference with the NF-κB family of transcription factors. NF-κB is crucial in regulating a number of genes important in both the inflammatory and antiviral response (Figure 6). NF-κB can be activated in response to numerous innate immunity signaling cascades, including tumour necrosis factor alpha (TNF-α) and pattern recognition receptors (PRRs). PRRs detect pathogen associated molecular patterns (PAMPs), such as foreign proteins or nucleic acids. This results in the activation of a coordinated signaling cascade, leading to the activation of the IKK complex. The phosphorylation and degradation of IκB allows the release and translocation of NF-κB to the nucleus, where it can activate gene transcription of proinflammatory cytokines and upregulation of late expression of type 1 interferons. The IKK complex is composed of two catalytic components (IKKα and IKKβ) and a non-catalytic regulator subunit, NF-κB essential modulator (NEMO). NEMO is a crucial component of the IKK complex, and acts as a molecular scaffold to recruit upstream signaling complexes [80].

Multiple viral proteins have been shown to interfere with NF-κB signaling [81], including hepatitis C virus (HCV) core protein and HPV E7, both of which inhibit IKK activation preventing IκB degradation [82,83]. Other viruses, such as cytomegalovirus (CMV) and molluscum contagiosum poxvirus, directly target the NEMO scaffold protein, which disrupts IKK activation [84,85]. Interestingly, SV40 ST has been shown to upregulate NF-κB activation in a PP2A dependent manner, although specific inflammatory targets such as IL-8 are downregulated by expression of SV40 ST [86].

MCPyV ST expression results in a marked decrease in genes associated with the innate immune repose, such as CCL20, IL-8, TANK and CXCL-9 [54]. Many of the genes downregulated upon MCPyV ST expression are those associated with the NF-κB pathway. ST is able to negatively regulate NF-κB mediated transcriptional activation of NF-κB responsive genes, through a previously undescribed interaction of ST with NEMO. This interaction specifically inhibits phosphorylation of IKKα/IKKβ, thus rendering NF-κB unable to translocate to the nucleus [54].

The poylomavirus ST-PP2A Aα interaction is well known. However MCPyV ST has also been found to interact with PP4C and/or PP2A Aβ, and this is thought to regulate NF-κB activation [54]. To date, it is unclear whether ST directly interacts with PP4C to promote dephosphorylation of this complex or if PP4C is part of a complex with ST and PP2A Aβ. Despite this uncertainty, it is clear that the activity of cellular phosphatases and their association with MCPyV ST is important for IKK complex dephosphorylation and NF-κB-mediated transcription.

MCPyV LT expression has also been found to play a role in downregulating the innate immune response. It inhibits Toll-like receptor 9 (TLR9) expression in epithelial and MCC cells [87]. TLRs are a subset of PRRs, and are an indispensable part of the innate immune response. TLR activation by PAMPs produces molecules for clearing infection, such as antimicrobial peptides, cytokines, and chemokines [88]. In particular, TLR9 senses viral or bacterial double-stranded DNA, as these contain many nonmethylated CpG motifs [89]. Importantly, when TLR9 recognises pathogenic DNA, it activates NF-κB in immune cells, leading to increased production of inflammatory molecules [90]. A number of double-stranded DNA oncogenic viruses, including HPV, inhibit the expression of TLR9 [91,92,93]. MCPyV LT also inhibits TLR9 expression. It does so by decreasing the mRNA levels of the C/EBPβ transactivator, which is a positive regulator of the TLR9 promoter. This leads to a greatly reduced binding of C/EBPβ to its response element on the TLR9 promoter. ST may also play a role in this, as its expression also appears to inhibit TLR9, but the mechanism is unknown. As such, LT targeting of C/EBPβ may therefore be important in establishing viral persistence [87]. These findings show that MCPyV has a complex and multifaceted defence against innate immunity. This allows the virus to persist, and may contribute to eventual tumourigenesis.

### 5.4. MCPyV Assembly and Egress

MCPyV virions tend to localise to the nucleus and the nuclear periphery prior to egress [94]. Apart from this, very little is known about MCPyV assembly and egress. It may be that LT-mediated sequestration of hVam6p to the cell nucleus might contribute to egress through lysosomal processing during virus replication [42]. Unlike other polyomaviruses, such as SV40, JCPyV and BKPyV, MCPyV does not encode an agnoprotein, which is known to be important in virus particle assembly and maturation [95]. In addition, it does not seem to encode an equivalent of the SV40 VP4, which has been shown to trigger lytic virion release [96]. Therefore, it must employ an alternative pathway.

In general, cell lysis is thought to be the primary mechanism of polyomavirus virion release, but accumulation of viral particles in SV40-infected cells, which subsequently shed, has also been observed [97]. One possibility is that, if the natural host of MCPyV is a type of skin cell, it may not require lysis for release, as the natural process of keratinocyte desquamation may serve this purpose. Polyomavirus assembly and egress is still poorly understood, and until a permissive cell culture model is developed for MCPyV, questions about the stages of the viral lifecycle cannot be fully addressed.

## 6. MCPyV and Tumourigenesis

**Figure 7:**
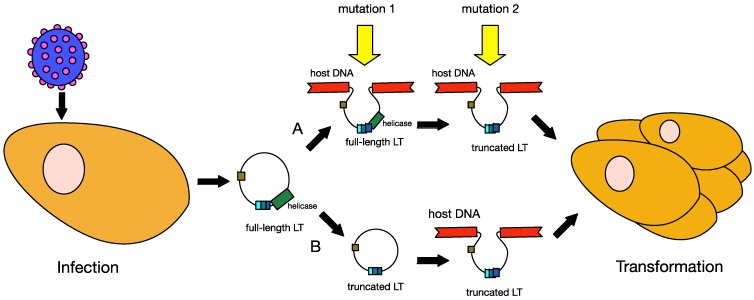
Models of MCPyV-induced MCC tumourigenesis. MCPyV infection is thought to occur early in childhood of most people. Before tumourigenesis can occur, loss immunosurveillance must lead to proliferation of the virus. At least two mutations are needed before MCPyV can transform cells. In model A, the first mutation is thought to be the integration of the full-length viral genome into host DNA, while the second mutation is the truncation of LT. In model B, truncation of LT is thought to occur before integration. Either way, these changes in the virus lead to cellular transformation and tumour proliferation.

MCPyV infection is common in the general population, and the virus is considered a component of normal skin flora. However, in a limited number of cases, MCPyV clonally integrates into a Merkel cell, which may initiate tumourigenesis (Figure 7). As a tumour virus, MCPyV is considered to be a direct carcinogen, as it expresses oncogenes that directly contribute to cell transformation. However, MCPyV-induced oncogenesis often requires the virus to become replication-defective, allowing cellular proliferation [3].

Old age and immunosuppression are both risk factors for MCC. This may in part be due to the loss of immunosurveillance against MCPyV, allowing the normally low levels of virus to rise. The presence of antibodies against the capsid proteins of MCPyV in patients with MCPyV-positive MCC suggests viraemia prior to tumour formation.

Upon MCPyV discovery, understanding of its oncogenic mode of action was initially drawn from established animal polyomavirus studies into the tumourigenic mechanisms associated with polyomavirus T antigens. However, recent years have seen an increase in the knowledge of MCPyV involvement in cellular transformation and the role that the MCPyV T antigens play in the viral life cycle. 

The vast majority of polyomavirus literature asserts that polyomavirus LT, rather than ST is the major oncogene. For example, SV40 LT has been shown to be the major transforming protein, with ST playing an accessory role in tumourigensis [98,99]. In the case of MCPyV, however, the tumourigenesis mechanism appears to be more complicated. MCC cells appear to be reliant upon T antigen expression as pan-T antigen knockdown in MCPyV positive cell lines triggered growth arrest and cell death [100]. This presents the question as to whether LT or ST has a dominant effect upon cellular transformation.

### 6.1. Truncation of MCPyV Large T Antigen

The signature feature of MCPyV-positive MCC is the presence of mutations in LT. These mutations prematurely truncate the LT protein at its carboxy-terminus, which leads to the loss of the domains associated with viral replication [20,101,102,103]. The sites of mutations are randomly distributed in the LT from different tumours, but the pRB binding site is consistently preserved. Thus, LT proteins expressed in different tumours vary in size, depending on the site of their mutations [104]. The truncated LT lack the OBD and helicase domains required for viral replication but retains the capacity to trigger host cell proliferation. This indicates a strong selective pressure within the MCC tumours to eliminate the viral replication capabilities, and retain only replication-deficient copies of MCPyV. Besides the mutations in LT, several groups have also reported mutations in the origin of replication and the VP1 gene in MCC-derived MCPyV genomic sequences that prevent efficient replication and progeny virion production, respectively [37,69,94,104]. Therefore, a two-step mutation process towards tumourigenesis has been suggested.

Upon MCPyV integration, the viral genome retains the full-length LT, capable of initiating host DNA replication. This unlicensed replication will result in replication fork collision and DNA breakage, which will lead to cytopathic cell death [103]. Thus a second mutation is needed to eliminate LT-initiated DNA replication for the cell to survive once MCPyV has integrated, and only a cell with both mutations is likely to develop into a tumour. The requirement for two separate events prior to tumourigenesis may help to explain why MCC is so rare [3]. It is also possible, however, that LT may first become truncated, with MCPyV going through rolling circle replication prior to integration [105]. There is some evidence for this alternative hypothesis. It has been shown that, upon exposure to UV radiation, expression of truncated LT leads to defective DNA repair and cell cycle arrest [106], and this increase in genomic instability may promote integration of the viral genome.

### 6.2. MCPyV Large T Antigen as an Oncogene

The MCPyV genome has been demonstrated to be clonally integrated in up to 97% of MCC samples [23,107]. Expression of MCV T antigens from the integrated genome contributes to transformation of host cells. LT DNA is present at an average of 5.2 copies per tumour cell and LT protein expression can be detected in the nuclei of these cells [108].

**Figure 8:**
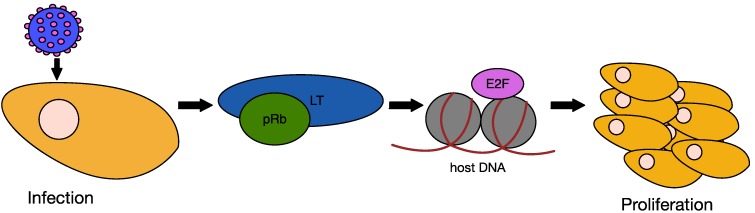
Effect of LT on cell proliferation. Upon MCPyV infection and T antigen expression, LT binds the regulatory protein pRb, thus inactivating it. This allows E2F to activate the transcription of cell cycle progression-associated genes, which switches the cell into S-phase and leads to cell proliferation.

In other oncogenic polyomaviruses, such as SV40, LT has been shown to be the major oncoprotein, with sT playing a lesser role. Overexpression of the SV40 LT is sufficient to transform mouse fibroblasts [109]. This transforming ability of LT is dependent upon manipulation of the key tumour suppressor proteins, p53 and Rb. The cell cycle checkpoint protein p53 is activated by cellular stress and blocks genome replication under conditions that could perpetuate DNA damage induced errors [110]. This is achieved by p53 promoting the expression of genes that induce DNA repair, cell-cycle arrest and apoptosis. Binding of SV40 LT to p53 inhibits this transcriptional activity and therefore permits inappropriate cellular proliferation [47]. The Rb protein controls entry into S-phase of the cell cycle. In resting cells pRb is bound to E2F, however upon activation of cyclin-dependent kinases pRb is phosphorylated, disrupting this complex. This permits E2F to activate the transcription of genes required for cell cycle progression. The LXCXE motif within LT mediates binding to Rb (Figure 8), which inhibits the interaction with E2F, thus bypassing this S-phase checkpoint. The DnaJ domain of LT also plays a role in this interaction, bringing together Hsp70 and Rb family members to facilitate the release of E2F in an ATP dependent fashion [111].

MCPyV LT is truncated in tumour cells, and one reason, as mentioned above, is to prevent unlicensed gene replication. However, the majority of reported LT mutations involve truncation of the entire C-terminal domain, as opposed to more subtle inactivating mutations. This suggests that there is an additional selective pressure upon transforming cells for removal of this region of LT. Consequently it has been shown that truncated LT is more efficient at inducing cellular proliferation than full length LT [112]. Moreover, overexpression of the LT carboxy-terminal 100 amino acids had an inhibitory effect upon the growth of certain cells types. Subsequent investigations have revealed that MCV infection activates the cellular DNA damage response in a manner dependent upon the LT carboxy-terminus [113]. Activation of DNA damage kinases was found to promote cell cycle arrest in a p53 dependent manner. Comparable to this, SV40 infection activates the ATM DNA damage pathway [114]. However, as the SV40 LT inhibits downstream activities of p53, cellular proliferation is not inhibited. Therefore LT truncations are likely selected to avoid activation of the DDR in the absence of p53 inactivation. In addition, LT has also been shown to increase levels of the cellular oncoprotein, survivin, which is dependent upon its Rb binding capacity [115]. Survivin is a member of the inhibitor-of-apoptosis proteins family, the overexpression of which is associated with numerous cancers [116].

With SV40 as the model, MCPyV LT would be expected to be the main viral oncoprotein driving cellular transformation. However, in contrast to SV40, neither full length, nor truncated MCPyV LT can initiate cellular transformation [74]. Some evidence suggests that MCPyV differs from other polyomaviruses in this respect, with ST the major oncogenic factor, which will be discussed below. One contributing factor to this diminished transformation ability of LT is that it does not appear to interact with p53, unlike the SV40 homologue [112]. This does not imply that LT does not play a role in MCPyV tumourigenesis, as it binds to both pRb and Hsc70, which are known to play a role in cell proliferation [37,103]. In addition, MCPyV LT does interfere with pRb-E2F binding, and is therefore capable of effecting cellular proliferation. In addition, recent data appear to support the role of LT as the major oncogenic factor, demonstrating that ectopic expression of LT alone could rescue pan-T antigen knockdown [117]. The relative importance of the small and large T antigens in MCC therefore requires further investigation.

### 6.3. MCPyV Small T Antigen as an Oncogene

There is new evidence that, unlike in other oncogenic polyomaviruses, MCPyV ST is as important for tumourigenesis, if not more so, than MCPyV LT. For example, it has been demonstrated that ST is more commonly expressed in MCC tumour samples than LT, indicating a significant role of ST in tumourigensis and tumour cell maintenance. In fact, 92% of MCC tumour samples are positive for ST expression, while only 75% are positive for LT. In addition, knockdown of ST in MCPyV-positive MCC cells inhibits growth [74]. Furthermore, expression of ST is sufficient to induce rodent fibroblast transformation, loss of contact inhibition, anchorage-independent and serum independent growth, while expression of LT does not lead to these changes [74]. These are key hallmarks of an oncogenic viral protein and confirm the important role that MCPyV ST plays in MCC tumourigenesis and pathology.

**Figure 9:**
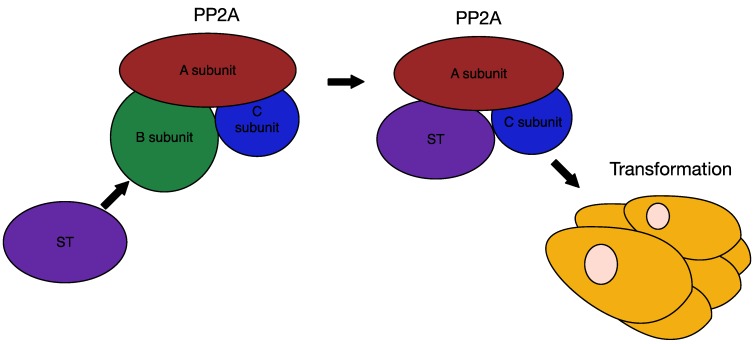
Interaction of ST with PP2A. Polyomavirus ST competes with the B subunit of PP2A for binding to the structural A subunit and the catalytic C subunit. In SV40, this interaction between ST and PP2A Aα leads to cell transformation. Although the interaction of MCPyV ST with PP2A Aα may not be necessary for transformation, interaction with PP2A Aβ or the related PP4C may play a role.

SV40 ST, being only an accessory oncogene, primarily contributes to cell transformation by targeting PP2A (Figure 9). This ability to bind PP2A is a common feature of polyomaviruses, as both SV40 ST and MPyV ST/MT bind PP2A [37]. Both MCPyV and SV40 ST bind the PP2A structural Aα subunit and the catalytic subunit thereby altering the substrate specificity of the PP2A holoenzyme [53,118]. SV40 is able to subvert the normal host phosphatase systems, as its ST binds to PP2A Aα in such a way that it overlaps the B subunit-binding domain [54,119]. This allows SV40 ST to compete with B subunit binding thereby inhibiting PP2A targeting and subsequent dephosphorylation of target substrates [98,120,121]. Alternatively, ST could act in a positive manner by redirecting PP2A phosphatase activity to alternative proteins [120].

Although MCPyV is related to SV40 and shares similar gene structure, it is becoming apparent that these two polyomaviruses differ drastically in their carcinogenic mechanisms. While the SV40 ST-PP2A Aα interaction is critical for SV40 induced transformation and cell proliferation, this may not be the case for MCPyV ST. MCPyV ST mutants with disrupted ST-PP2A Aα interaction were fully capable of inducing both rodent cell transformation and anchorage-independent colony formation [74]. This clearly indicates that while these viruses may contain similar protein structure and binding domains, their mode of action is distinct. If MCPyV ST oncogenicity does not depend upon its ability to bind PP2A Aα, another mechanism must be in place, perhaps through binding to PP2A Aβ and/or PP4C. It has been demonstrated that SV40 ST activates the Akt pathway by preventing PP2A Aα mediated dephosphorylation of Akt [122,123]. However, MCPyV ST has little effect at that point in the AkT-mTOR pathway, instead acting downstream of mTOR [74].

The PI3K-AkT-mTOR signalling pathway plays a significant role in the regulation of cap-dependent translation in tumourigenesis [124,125,126]. An important step in cap-dependent translation is the binding of the eukaryotic translation initiation factor 4E (eIF4E) to specific mRNA molecules [127]. Assembly of eIF4E at the mRNA cap as part of the multisubunit eIF4F complex initiates ribosome recruitment. A major regulator of this pathway is the eukaryotic translation initiation factor 4E-binding protein 1 (4E-BP1), which sequesters eIF4F to prevent eIF4F complex formation [128]. Interference with this pathway is important for tumour cell growth, as overexpression of eIF4F can induce rodent cell transformation [129,130]. Upon 4E-BP1 phosphorylation by mammalian target of rapamycin (mTOR) 4E-BP1 releases eIF4F, allowing cap assembly and cap-dependent translation [131]. 

MCPyV ST acts by reducing the turnover of hyperphosphorylated 4E-BP1, which increases eIF4F activity, and, in turn, cap-dependent translation. Moreover, expression of a constitutively active mutant 4E-BP1 protein reverses ST induced cell transformation and is independent of PP2A Aα and Hsc70 binding [74]. This demonstrates the importance of ST in regulating cap dependent translation, and that through this mechanism ST is able to contribute to MCC tumourigenesis. In contrast, it has been demonstrated that SV40 ST diminishes 4E-BP1 phosphorylation [132], which is another interesting difference to the established view that polyomavirus ST tumorigenic ability is intrinsically linked to PP2A Aα binding.

Interestingly, the newly-discovered LSD domain of MCPyV ST is not only important for stabilising LT and viral genome replication, but may be required for rodent cell transformation. A mutation within the LSD region ablates ST ability to induce transformation [55]. This mutation does not affect ST ability to bind PP2A, once again confirming that the ability of ST to induce transformation is independent of PP2A, and is distinct from other polyomaviruses in this respect.

However, at present there seems to be conflicting view points as to the tumorigenic nature of MCPyV ST. While expression levels of ST in MCC cell lines and MCC tissues appear to be low [117], ST is more commonly detected in MCC tissues than LT in immunohistochemistry analysis, and knocking out ST in MCPyV-positive MCC inhibits cell growth [74]. Furthermore, as discussed above, there is some controversy as to which of the two proteins is most important for cancer cell growth and proliferation. It may be that ST is essential for initial transformation, while LT is necessary for subsequent survival and proliferation.

It is clear that there is a degree of uncertainty that needs to be addressed. Regardless, there is a large robust body of evidence that indicates that MCPyV ST is crucial to tumourigensis and maintenance of the tumour state. Indeed, the fact that MCPyV is the only human polyomavirus associated with tumourigenesis indicates that there are some aspects of this virus that do not follow the normal polyomavirus stereotype, and that there likely are novel mechanisms that set it apart from its relatives.

## 7. Therapies for MCC

Conventional treatment of MCC depends on the disease stage. The standard therapy for localised tumours is surgical excision with 2–3 cm margins. If regional lymph nodes are thought to be involved, dissection is undertaken. In most cases, adjuvant radiotherapy to the tumour site and, if necessary, regional lymph node site, is given. This has been associated with decreased risk of regional relapse [133]. If a tumour cannot be excised, radiation on its own can be performed, as MCC is a relatively radiosensitive tumour [134]. If MCC has metastasised, various regimens of broad-spectrum chemotherapy have been used, such as anthracyclines, cyclophosphamide, etoposide, and platinum derivatives, alone or in combination. Over half of MCC patients respond to chemotherapy, but prognosis is poor, and the median survival is 21.5 months [135].

Previous to the discovery of MCPyV as the causative agent of the majority of MCC cases, the molecular basis of the cancer was not well researched. Therefore, only generic therapies are available for treatment. However, this is likely to change as more is uncovered about MCPyV tumourigenesis and drug targets are identified. MCPyV T antigen expression is necessary for survival of MCPyV-positive MCC cells, therefore LT and ST are considered to be potential therapeutic targets.

Some preliminary drugs inhibiting MCPyV-positive MCC have been identified *in vitro*. For example, type I interferon (IFN) reduces LT expression and inhibits cell viability in MpyV-positive MCC cell lines [136]. However, IFN treatment failed to induce a clinical response in two patients with late-stage MCC [137]. On the other hand, YM155, a drug inhibiting surivin expression, which is upregulated by LT and is important for the surivival of MCPyV-positive MCC cell lines, is cytotoxic in the nanomolar range and shows a cytostatic effect in MCC xenograft tumours in mice [115,138]. The most promising drug at the moment, however, is the small-molecule tyrosine kinase inhibitor, pazopanib [139], currently in a phase II clinical trial. The field remains open for novel drugs, especially those that target the newly-discovered effects MCPyV T antigens have on the innate immune response. In addition, inhibiting cap-dependent translation and mTOR signalling may also be beneficial in treating MCC.

MCC, like other virus-induced cancers, has viral antigens in its cells, and is thus immunologically responsive. In fact, improved immunosurveillance is likely behind the occasional spontaneous regression of disseminated MCC tumours [140]. Immunity-based approaches to treating MCPyV-positive MCC are promising, and work in this area is ongoing. Specific T lymphocytes that recognise various MCPyV epitopes have been identified, both within tumour cells and in the blood of both MCC patients and healthy test subjects [141]. 58 CD8+ T lymphocytes against 35 MCPyV T antigen epitopes have been identified, and these responses are present only in MCC patients [142]. Despite these specific T lymphocyte responses, MCC tumours persist and grow. This suggests that MCC tumours inhibit T lymphocyte cytotoxicity or otherwise evade the immune response. Nevertheless, DNA vaccines are a possibility, and preliminary work using murine tumour models to create vaccines has shown that it is possible to that target both ST and LT and elicit both CD4+ and CD8+ T lymphocyte response [143,144]. In addition, MCPyV-specific CD8+ T lymphocytes exhibit targetable exhaustion markers PD-1 and Tim-3 [145]. These markers are associated with reversible T-lymphocyte dysfunction, thus investigating antagonists of the PD-1 and Tim-3 pathways may yield additional MCC treatment measures.

Merkel cell carcinoma is highly metastatic, and designing drugs to inhibit this property would limit the mortality rate of late-stage disease. Uncovering the mechanism of what drives MCC metastasis is therefore of paramount importance. Determining if and how one or both of the oncoproteins of MCPyV induces cell motility and invasion will allow for the rational design of inhibitors to interfere with this mechanism.

## 8. Conclusions

From our understanding of MCPyV, a broad model of tumourigenesis can be presented. The virus is acquired by the advent of adulthood. The route of transmission is not yet known, as the virus is part of the microflora of the skin, it is likely that infection is cutaneous. Immunosupression, whether in the form of aging, HIV-AIDS, drugs, or UV damage, leads to loss of immunosurveillance and MCPyV reactivation. It is at that time that the mutations associated with MCPyV tumourigenesis can occur. It may be that UV or other types of external stimuli contribute to the likelihood of mutagenesis.

Regardless of the order of the two mutations, cells which have an integrated copy of replication-defective MCPyV display viral oncogene expression, which leads to clonal expansion, neoplastic progression, and, ultimately, development of MCC.

Each year, more studies shed light on the lifecycle and the tumourigenic potential of MCPyV. There is a mechanism for tumourigenesis, although it is not yet fully determined. In addition, details about the natural lifecycle of the virus are coming into light, such as attachment steps and the mechanism of replication. However, until a fully permissive cell culture model is developed, it will not be possible to fully elucidate the entry, replication, and egress of MCPyV.

There is some evidence that MCC might not be the only cancer MCPyV plays a role in, although the findings are, as yet, inconclusive. Chronic lymphocytic leukaemia (CLL) is one cancer where replication-deficient MCPyV has been isolated from a subset of tumours [146]. In addition, patients with MCPyV-positive MCC have a greater risk of developing CLL than patients with MCPyV-negative MCC [147]. CLL tumours in general do not reveal a pattern of MCPyV presence [25,148], although this does not preclude the virus from playing a role in a fraction of CLL cases. Another cancer where MCPyV might play a role is squamous cell carcinoma (SCC). Although some reports find no significant association [145,149], others stress that MCPyV DNA is present in 40% of cutaneous SCCs, which significantly correlates with MCPyV seropositivty [150]. In addition, nearly all MCPyV-positive SCCs have mutations within exon 2 that could produce a truncated version of LT [151]. Finally, a full-length MCPyV genome has been isolated from a Kaposi's sarcoma, and this genome expresses a truncated LT [152]. Thus MCPyV could have a role to play in the development of a number of cancers. 

MCC is a highly invasive and metastatic cancer. The reason behind these features has not been elucidated, but it may be that the T antigens are involved in promoting the characteristic, aggressive metastasis of MCPyV-transformed cells. Furthermore, the T antigens could have functions in such areas as lipid metabolism, the cell cycle, and DNA damage and repair. At the moment, knowledge of how LT and ST influence the multitude of cancer pathways is limited, and drawn mainly from work with SV40, despite mounting evidence that many mechanisms MCPyV LT and ST employ are divergent. A growing number of interactions between LT, ST, and host factors are being uncovered, but, undoubtedly, there is still much more left to find.

In order to understand both MCPyV as a virus and how it causes cellular transformation, and ultimately cancer, much more work needs to be done on the molecular virology of the virus, on the cellular biology of MCPyV-transformed cells and on the immunology of MCC. It has come to light that MCPyV has many unique features, and detailing further its molecular virology may uncover more important mechanisms of transformation, carcinogenesis, and cancer spread.
